# Cord blood T regulatory cells synergize with ruxolitinib to improve GVHD outcomes

**DOI:** 10.3389/frtra.2024.1448650

**Published:** 2024-12-11

**Authors:** Ke Zeng, Hongbing Ma, Meixian Huang, Mi-Ae Lyu, Tara Sadeghi, Christopher R. Flowers, Simrit Parmar

**Affiliations:** ^1^ Department of Lymphoma/Myeloma, The University of Texas at MD Anderson Cancer Center, Houston, TX, United States; ^2^ Department of Hematology, West China Hospital, Sichuan University, Chengdu, China; ^3^ Cellenkos Inc., Houston, TX, United States; ^4^ Department of Microbial Pathogenesis & Immunology, Texas A&M University, Bryan, TX, United States

**Keywords:** GVHD, T regulatory cell, allogeneic, cord blood, ruxolitinib

## Abstract

**Background:**

Adoptive therapy with umbilical cord blood (UCB) T-regulatory (Treg) cells can prevent graft vs. host disease (GVHD). We hypothesize that UCB Tregs can treat GVHD and synergize with ruxolitinib, Jak2 inhibitor, to improve outcomes.

**Methods:**

UCB Treg potency and efficacy was examined using cell suppression assay and xenogeneic GVHD model, respectively. Ruxolitinib was fed continuously in presence or absence of CellTraceViolet tagged UCB Tregs on days +4, +7, +11, +18. Mice were followed for survival, GVHD score, hematology parameters and inflammation.

**Results:**

Addition of ruxolitinib to UCB Tregs exerted synergistic suppressor function *in vitro* and improved persistence of UCB Tregs *in vivo*. Lower GVHD score, improved survival, increased hemoglobin level and platelet count, decreased inflammatory cytokines and decrease in CD3^+^ T cell lung infiltrate was observed in UCB Tregs+ruxolitinib recipients.

**Conclusion:**

UCB Treg+Ruxolitinib combination improves outcomes in xenogeneic GVHD and should be explored in a clinical setting.

## Introduction

Graft vs. host disease (GVHD) is a fatal complication of allogeneic stem cell transplantation, driven by donor derived T cell proliferation and characterized by excessive inflammation which can lead to widespread tissue injury and wasting phenomenon (1). CD4^+^CD25^+^FoxP3^+^ regulatory T cells (Treg) are a subset of T cells that regulate immune function and resolve unwanted inflammation (2). Tregs have been extensively studied for prevention and treatment of GVHD (3–5), with promising clinical results (6–11).

We have previously shown that Tregs derived from umbilical cord blood (UCB) co-express CD45RA^+^CD45RO^+^ (12) that allow for sustained *in vivo* proliferation of the injected cells; as well as retain their suppressor function in presence of dexamethasone (12, 13). Additionally, treatment with multiple injections of UCB Tregs can reduce burden of inflammation without interfering in the anti-tumor activity of CD19 CART cells in a xenogeneic lymphoma model (14). Recently, Kadia et al., showed that a single infusion of CK0801 Tregs can lead to durable independence from blood and platelet transfusion in patients with bone marrow failure (15). In a randomized placebo control trial, multiple infusions of CK0802 Tregs led to improvement in survival in patients with COVID associated acute respiratory distress syndrome (13). Combination of donor derived Tregs and ruxolitinib, a Jak2 inhibitor currently approved agent for steroid refractory GVHD (16), has been shown to exert synergistic effect to improve GVHD outcomes without interference in graft vs. leukemia effect (17). We hypothesize that addition of UCB Tregs to ruxolitinib can improve results in GVHD.

## Methods

### UCB Treg cell generation and function

UCB derived Tregs were generated as described previously (12, 18) and/or provided by Cellenkos® Inc. (Houston, TX, USA). Cell characterization was performed as described in Supplementary Material. Cell suppression assay was performed as described previously (14).

### *In vivo* GVHD model

Animal procedures were performed according to an approved protocol by The University of Texas MD Anderson Cancer Center Institutional Animal Care and Use Committee. Xenogeneic GVHD model was established as described previously (4). CellTraceViolet (CTV) dye labeled UCB Tregs (1 × 10^7^ cells) were injected on days +3, +10, +17, and +24. Mice received 1 mg ruxolitinib daily by oral gavage for 14 consecutive days. Mice were monitored and weighed twice weekly. GVHD score was calculated as described previously (Supplementary Table S1) (19). Peripheral Blood (PB) from mice was collected weekly and at euthanasia, for flow analysis. Euthanasia was performed according to institutional guidelines. Room air in the mice chamber was gradually replaced by 100% CO_2_, from a compressed gas cylinder at a flow rate that displaced 30%–70% of the chamber volume per minute until the concentration of CO_2_ reached 100%. Upon achieving this concentration, the mice remained in the chamber for at least, an additional three minutes to ensure effective euthanasia. Plasma was analyzed for inflammatory cytokines using Human Cytokine 42-plex Discovery Assay Kit (Eve Technologies, Calgary, Canada). Organs of xenografts were harvested, homogenized, and analyzed as described previously (14).

### Statistical analysis

All statistical analyses were done with GraphPad Prism 9 software (San Diego, CA, US). Data are presented as mean ± SEM. *P*-values were calculated using 2-tailed *t*-test with 95% confidence interval, one-way ANOVA, or two-way ANOVA for evaluation of statistical significance compared with the untreated controls. *P* < 0.05 was considered statistically significant.

## Results and discussion

### UCB Tregs can treat GVHD without affecting GVL

**Figure 1:**
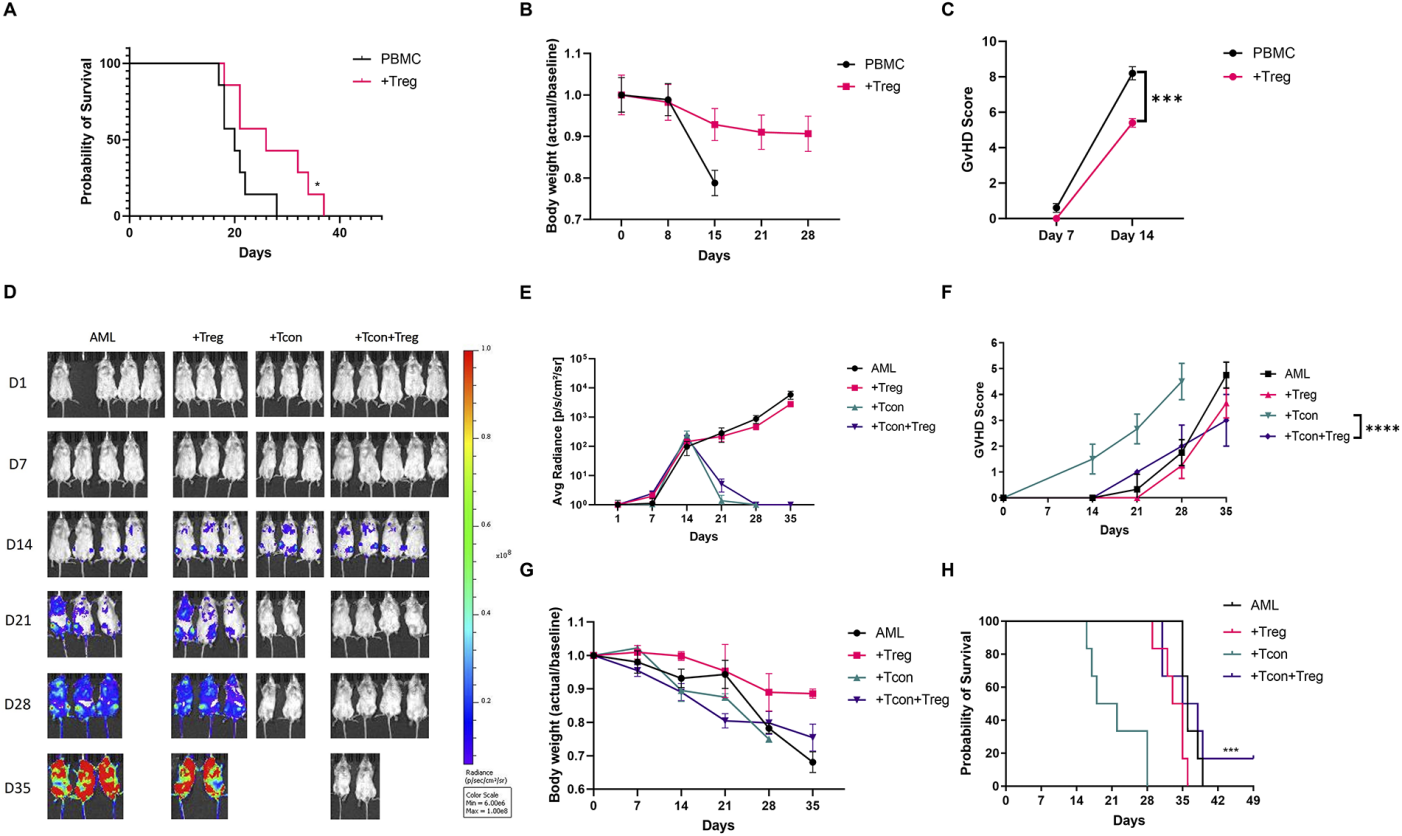
UCB Tregs can treat GVHD and preserve GVL. Xenogeneic GVHD model using female Rag2-γc- mice, that underwent sublethal irradiation on day-1 and received tail vein injection of 10^7^ human peripheral blood mononuclear cells (PBMCs) on Day 0. In the treatment arm, 10^7^ UCB Treg cells were injected through tail vein on days +4, +11, +18, and +25. *N* = 6 each arm. **(A)** UCB Tregs improve survival compared to control. **(B)** UCB Tregs maintain body weight compared to continued weight loss in control arm. **(C)** UCB Tregs significantly improve GVHD scores, from days 7 and 14 compared to control groups. Graft vs. Leukemia (GVL) xenogeneic model was set up using female Rag2-γc- mice, that underwent sublethal irradiation, followed by 3 × 10^6^ GFP-labeled HL60 AML cells in presence or absence of by 3 × 10^6^ UCB Tregs on Day 0, followed by tail vein injection of 3 × 10^6^ Tcon cells. Treatment groups were divided into: Arm 1: HL60 alone (AML); Arm 2: HL60 + UCB Tregs (+Treg); Arm 3: HL60+Tcon (+Tcon); Arm 4: HL60+UCB Tregs+Tcon (+Tcon+Treg), *N* = 6 each arm. **(D)** UCB Tregs do not increase tumor burden in GVL model. HL60 tumor burden in mice was evaluated by Non-invasive bioluminescence imaging (BLI) with the IVIS Lumina X5 Imaging System. Ventral images are shown for each day of capture. Non-invasive BLI showed clear evidence of disease progression in control arm, AML and +Treg. No evidence of disease in +Tcon and +Tcon+Tregs recipients on day 21 and day 28. **(E)** Quantitative analysis of the BLI measurements (photons/sec/cm^2^/sr) showed no differences in the +Tcon and +Tcon+Tregs recipients by day 35. **(F)** UCB Tregs improve GVHD score in GVL model. Significant improvement in GVHD scores in +Treg+Tcon arm compared to +Tcon arm alone. Scores were evaluated bi-weekly, with statistical analysis conducted using data from day 28 to compare the different arms. **(G)** UCB Tregs maintain weight in GVL model. No differences observed between +Tcon vs. +Tcon+UCB Treg arm. Weight measured twice weekly, and results presented as fold changes compared to baseline. **(H)** UCB Tregs do not compromise survival in GVL. Over a 49 day follow up, +Tcon+UCB Treg arm shows survival benefit. Addition of UCB Tregs to AML did not have any significant impact on tumor burden. Error bars represent SEM. Statistical differences compared with control were quantified by one-way ANOVA or paired student's *t*-test; *p < 0.05, **p < 0.01, ***p < 0.001, ****p < 0.0001, ns, not significant; SEM, standard error of means.

*Ex vivo* expanded UCB Tregs express CD4^+^CD25^+^CD127^lo^FOXP3^hi^Helios^hi^ phenotype (Supplementary Figure S1) and have been shown to retain their function in presence of dexamethasone (12). In an established haploidentical murine GVHD model, donor Tregs injection on day +11 led to improvement in survival and decrease in GVHD score (20). In an exploratory study, two out of five patients suffering from chronic GVHD, who received donor derived, *ex vivo* expanded, Treg cells at a median of 35 months after their allogeneic hematopoietic cell transplantation showed improvement in their condition which allowed decrease in their steroid intake and the other three patients had stable disease (21). To evaluate whether UCB Tregs can treat established GVHD in a completely mismatched setting, we injected multiple of UCB Tregs, in a fixed dose of 10 million cells, in a xenogeneic GVHD model (Supplementary Figure S2). As shown in Figure 1A, UCB Treg cell treatment led to improvement in survival; preservation of weight (Figure 1B) and decrease in GVHD score (Figure 1C). A major concern exists in regard to impact of any GVHD treatment modality on possibly interfering in the donor T cell mediated graft vs. leukemia (GVL) effect (22). To evaluate the impact of UCB Tregs on GVL, 3 × 10^6^ GFP labeled HL60 leukemia cells were injected through tail vein into sublethal irradiated non-SCID gamma null (NSG) mice to establish acute leukemia. On day +1, mice were injected with 3 × 10^6^ CD4^+^25^−^ Tcon cells in presence or absence of 3 × 10^6^ UCB Tregs injected on day +4 (Supplementary Figure S2). No evidence of leukemia was detected in Tcon and UCB Tregs + Tcon recipients by day +28 by non-invasive bioluminescence imaging (Figures 1D,E). However, all Tcon recipients succumbed to GVHD without evidence of leukemia by day +35 (Figure 1D). Whereas highest GVHD score was observed in Tcon recipients, addition of UCB Tregs reduced it significantly over 35 days (*p* < 0.0001; Figure 1F). While body weight was comparable between Tcon and Tcon + UCB Treg recipients (Figure 1G), a significantly superior survival was observed in the latter arm (*p* < 0.001; Figure 1H). Our findings support that UCB Tregs can treat xenogeneic GVHD without compromising GVL effect. Similar uncoupling effect has been reported in a xenogeneic lymphoma model, where UCB Tregs dampened systemic inflammation without interfering in the on-target anti-tumor effect of CD19 CART cells (14). Long term follow up of multiple clinical studies examining Treg cells in GVHD, also do not report any detrimental effect of Tregs on the risk of leukemia relapse (6–10, 23). Thus, adoptive therapy with UCB Tregs presents itself as a viable strategy for treatment of GVHD.

### UCB Tregs synergize with ruxolitinib to improve GVHD outcomes

**Figure 2:**
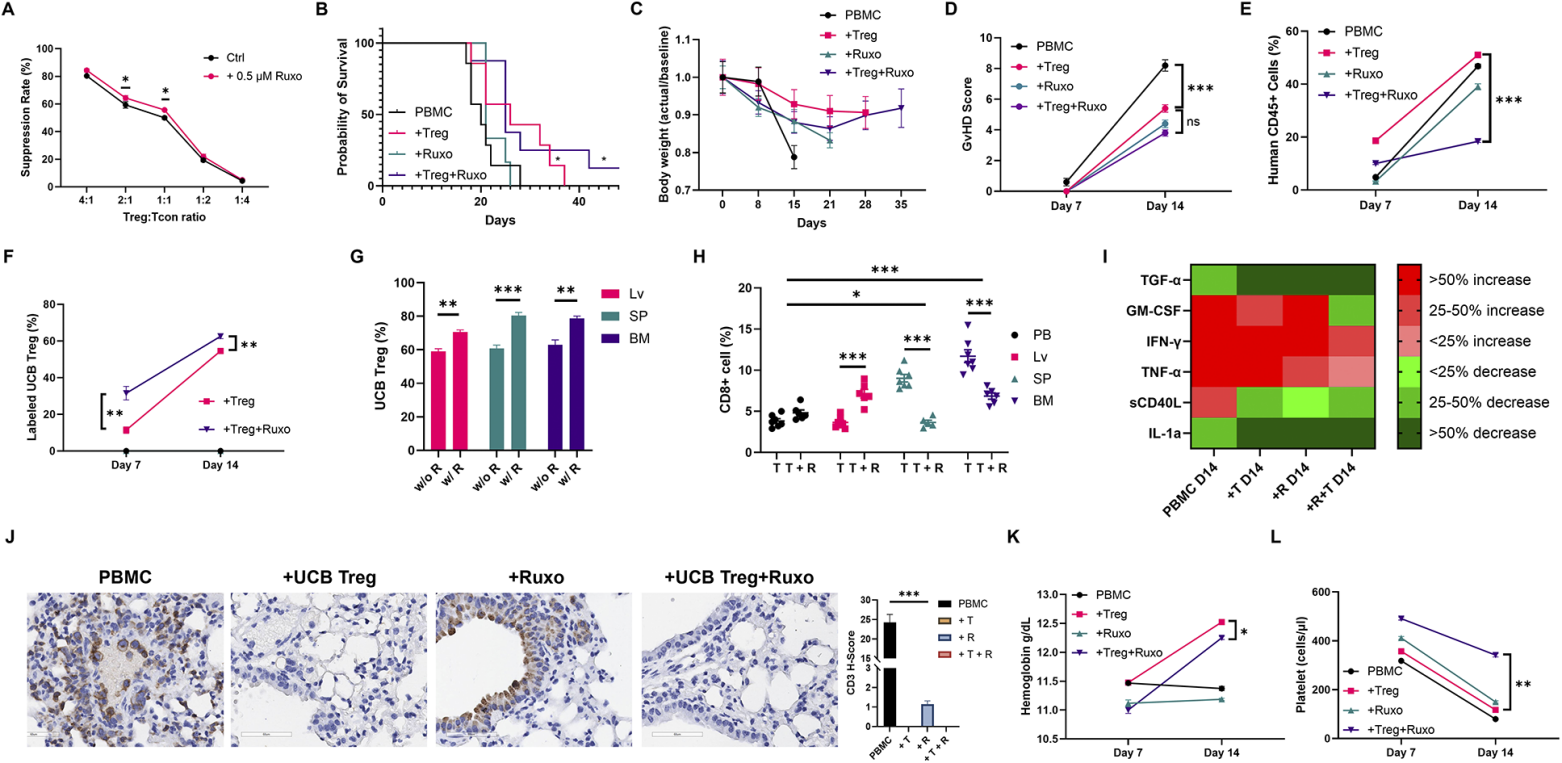
UCB tregs synergize with ruxolitinib to improve GVHD outcomes. **(A)** Ruxolitinib improves UCB Treg suppression. UCB Tregs co-cultured with CTV-labeled Tcon (CD4^+^CD25^−^) cells at different ratios of 4:1, 2:1, 1:1, 1:2, and 1:4 in the presence of CD3/28 beads. Proliferation of CTV-labeled Tcons was assessed by the LSR Fortessa Cell Analyzer after 96 h of culture. Percentage suppression was calculated using the following formula: 100% × (1−percentage of proliferating CTV-diluting Tcons in the presence of UCB Tregs at a different ratio/percentage of proliferating CTV-diluting Tcons when cultured alone). Ruxolitinib (0.5 μM) led to improvement in UCB Treg suppression at 2:1 and 1:1 Treg: Tcon ratio. Impact of addition of ruxolitinib at different UCB Tregs: Tcon ratios was: 4:1 (84.0% vs. 80.2%); 2:1 (64.1% vs. 59.3%); 1:1 (55.4% vs. 49.1%); 1:2 (22.4% vs. 19.1%); 1:4 (5.4% vs. 4.1%), respectively. Xenogeneic GVHD model was established using female Rag2-γc- mice, that received tail vein injection of 10^7^ human PBMCs on Day 0. In the treatment arm, mice received 1 mg of Ruxolitinib (45 mg/kg) daily orally from day −1 until day 14. Additionally, 10^7^ UCB Tregs were injected by tail vein on days +4, +11, +18, and +25. Arm 1: control (PBMC); Arm 2: PBMC+UCB Tregs (+Treg); Arm 3: PBMC+ruxolitinib (+Ruxo); Arm 4: PBMC+UCB Tregs+ruxolitinib (+Treg+Ruxo). *N* = 6 each arm. **(B)** UCB Tregs improve survival in xenogeneic GVHD. When compared to control arm, PBMC, significant improvement in survival was seen in +Treg and +Treg+Ruxo arms, at a follow up of 42 days. **(C)** UCB Tregs maintain body weight in xenogeneic GVHD. Weights were recorded twice a week, and the data are presented as fold changes over successive days relative to the baseline. **(D)** UCB Tregs lower GVHD score. When compared to control arm, +Treg, +Ruxo and +Treg+Ruxo led to significantly lower GVHD score at day 14. The scoring was performed bi-weekly. **(E)** UCB Tregs synergize with ruxolitinib to decrease circulating human CD45^+^ T cells (gated on live cells) at day 14 in xenogeneic GVHD. **(F)** Ruxolitinib increases UCB Tregs persistence. Significantly higher circulating CTV labeled Tregs on day 7 (*p* < 0.01) and day 14 (*p* < 0.001) in +Treg+Ruxo vs. +Treg arm, respectively. **(G)** Ruxolitinib increases UCB Tregs tissue persistence. Flow cytometric analysis of harvested organ cell suspensions at euthanasia on day 14 showed higher level of CTV labeled Tregs in UCB Tregs+ruxolitinib (w R) recipients compared to UCB Tregs recipients (w/o R), across different lymphoid sites, including spleen (SP), liver (LV) and bone marrow (BM). **(H)** Ruxolitinib addition to UCB Tregs leads to decrease in CD8^+^ T cells in spleen and bone marrow in xenogeneic GVHD at day 14. *Y*-axis shows CD8^+^ T cells gated on human CD45^+^ cells. T = UCB Tregs; T + R = UCB Tregs + ruxolitinib. Compared to peripheral blood (PB), proportion of CD8^+^ cells was significantly higher in the spleen (SP) (*p* < 0.05) and bone marrow (BM) *p* < 0.001. Addition of ruxolitinib led to a significant decrease of CD8^+^ cells in spleen and bone marrow and an increase in liver (*p* < 0.001). **(I)** UCB Tregs and ruxolitinib decrease systemic inflammation in xenogeneic GVHD. On day 7 and 14, plasma cytokines level was quantified and analyzed. Cytokines at day 14 were normalized to day 7. The categorical heatmap shows the percentage changes in cytokine levels at day 14 compared to day 7 for TGF-α, GM-CSF, IFN-γ, TNF-α, sCD40l, and IL-1a. Changes are color-coded: shades of green indicate decreases (less than 25%, 25%-50%, more than 50%) and shades of red indicate increases (less than 25%, 25%-50%, more than 50%). **(J)**. Histopathologic examinations of lung at 40× magnification, shows tissue destruction and high CD3^+^ staining in the control PBMC arm. Tissue architecture is somewhat preserved in PBMC+ruxolitinib arm, with high concentration of CD3^+^ staining in the alveolar lining as well as in the parenchyma. Complete resolution of CD3^+^ infiltrate as well as tissue architecture preservation is seen in UCB Treg recipients with or without ruxolitinib. Quantification analysis of the H-score for human CD3 positivity (right panel). The H-score was defined by the percentage of strongly positive stain × 3 + moderately positive stain × 2 + weakly positive stain × 1. A final value of 0–300 was also calculated at 40× magnification using the software HALO (v3.5-3,577.140). A *p* < 0.05 was considered statistically significant. *p < 0.05, **p < 0.01, ***p < 0.001. **(K)**, UCB Tregs improve hemoglobin in ruxolitinib recipients. On day 14, ruxolitinib recipients show lower hemoglobin levels compared to UCB Tregs recipients. Addition of UCB Tregs to ruxolitinib increases day 14 hemoglobin level. **(L)** UCB Tregs improve platelet decrease in ruxolitinib recipients. Compared to day 7, decrease in platelet counts observed in all arms on day 14. UCB Tregs + ruxolitinib recipients preserved their platelet count. The statistical differences were quantified by a one-way ANOVA or student's *t*-test. Error bars represent SEM (*n* = 7); statistical differences compared with the control arm were quantified by one-way or two-way ANOVA using GraphPad Prism software: *p < 0.05, **p < 0.01, ***p < 0.001.

Next, we proceeded to examine whether UCB Tregs can be added to ruxolitinib, an approved agent for treatment of steroid refractory GVHD (16). In a recent study of 35 patients with aplastic anemia, prophylatic administration of ruxolitinib significantly lowered incidence of moderate to severe acute GVHD (17.1% vs. 48.6%) (24). However, since ruxolitinib can suppress both T cells (25) and Tregs (26) and is accompanied by hematologic toxicity including anemia and thrombocytopenia (16) that can be dose limiting in GVHD treatment, we examined whether the addition of UCB Tregs can mitigate such a side effect. We analyzed the proliferation of CTV labeled CD4^+^25^−^ Tcon cells cultured with different ratios of UCB Tregs in presence or absence of ruxolitinib. A slight, but significant improvement in the cell suppression was observed with the addition of ruxolitinib at UCB Treg:Tcon ratio of 2:1 and 1:1 (*p* < 0.01; Figure 2A). Similar synergistic effect of 0.1 um ruxolitinib on cell suppression function of human Treg cells has been reported at 120 h of co-culture, where superior survival was observed in recipients of ruxolitinib and Tregs compared to either agent alone (17). To understand whether a comparable effect will be recapitulated in a xenogeneic GVHD model, ruxolitinib and UCB Treg treatment combination was examined (Supplementary Figure S2). Improvement of survival was observed in the UCB Tregs + ruxolitinib recipients compared to all other arms (Figure 2B), which was accompanied with preservation of body weight (Figure 2C) and lowering of GVHD score (Figure 2D). When gated on live cells, while the circulating human CD45^+^ cells in the peripheral blood increased over time, a significantly lower level was observed in the UCB Tregs + ruxolitinib recipients when compared to UCB Tregs alone recipients at day +14 (*p* < 0.001; Figure 2E). This synergistic effect might be due to engagement of complimentary pathways since the effects of ruxolitinib on Tregs are immune-context dependent (27).

To study their *in vivo* distribution pattern, UCB Tregs, were labeled with CTV, a low-cytotoxicity intracellular dye, that is detectable for at least seven days post-labeling. As shown in Supplementary Figure S3, when gated on human lymphocytes, nearly all the CD4^+^25^+^127^lo^ Treg cells comprised of CTV-labeled cells. On day +14, CTV labelled UCB Tregs percentage was significantly higher in the UCB Tregs + ruxolitinib vs. UCB Tregs alone recipients in peripheral blood (PB) (Figure 2F), and in liver, spleen and bone marrow (Figure 2G). When gated on human CD45^+^ cells, CD8^+^ T cells percentages decreased in spleen and bone marrow (Figure 2H). Cytotoxic CD8^+^ T cells play a pivotal role in the pathogenesis of acute GVHD because they directly attack nonmalignant host tissues through effector molecules (28), and therefore, their decrease at the level of the target tissue in the UCB Tregs recipients, alone and in combination with ruxolitinib, highlights an important mechanism deployed by UCB Tregs to resolve GVHD. Although a relative increase in CD8^+^ T cells was observed by flow cytometry in the liver, such an increase was not reflected on the immunohistology studies (Supplementary Figure S5).

To further examine whether addition of UCB Tregs has an impact on tissue infiltration with CD3^+^ lymphocytes, IHC staining was performed and quantified using H-score (12). In xenogeneic GVHD model, lung has been identified as a target organ for immune mediated tissue destruction (29). IHC section of lung histologic section showed a decrease in CD3^+^ staining in ruxolitinib recipients, with a complete resolution of CD3^+^ infiltrate in UCB Tregs and in UCB Tregs + ruxolitinib recipients. Quantification of the CD3^+^ cell infiltrate using H-score mirrored the histology findings, which shows that UCB Tregs decrease the T-cell mediated tissue damage in GVHD. Histology examination of spleen and liver is shown in Supplementary Figure S5. Furthermore, a corresponding decrease in the inflammatory cytokines, including GM-CSF, IFNγ, TNFα, sCD40l, IL-1a, observed in the treatment arms at day +14 (Figure 2I), underscores the multiple mechanisms at play for dampening GVHD (30).

Since ruxolitinib is associated with grade 3 and/or 4 thrombocytopenia in steroid refractory GVHD (16), we sought to examine whether UCB Tregs has an impact on this drug toxicity. As shown in Figure 2L, on day 14, platelet count decrease was lesser in UCB Tregs + ruxolitinib recipients when compared to ruxolitinib alone or UCB Tregs alone recipients. Furthermore, an improvement in the hemoglobin levels was observed in the UCB Tregs + ruxolitinib recipients when compared to ruxolitinib alone recipients (Figure 2K). In addition to a possible direct protective effect on the bone marrow, the improvement in cytopenias might be attributed to the relief from IFNγ mediated bone marrow suppression which is reversed by the UCB Tregs (31).

We conclude that UCB Tregs synergize with ruxolitinib to treat xenogeneic GVHD through multiple mechanisms and lead to improve outcomes. This combination should be examined in a clinical setting.

## Data Availability

The original contributions presented in the study are included in the article/Supplementary Material, further inquiries can be directed to the corresponding author.
