# Supplementary material for: Cord blood T regulatory cells synergize with ruxolitinib to improve GVHD outcomes
Source: Front Transplant. 2024 Dec 11;3:1448650. doi: 10.3389/frtra.2024.1448650 (PMC11668690; doi:10.3389/frtra.2024.1448650)
Supplement: Supplementary file 1 [file Datasheet1.pdf]

Supplementary Material

Cord Blood T Regulatory Cells Synergize with Ruxolitinib to Improve GVHD Outcomes

Table of Figures

Figure S1. **Phenotypic Characterization of Ex Vivo Expanded UCB Tregs** 3
Figure S2. **Xenogeneic GVHD model for Ruxolitinib and UCB Tregs** 4
Figure S3. **Impact of UCB Tregs on Xenogeneic GVL** 5
Figure S4. **CellTrace^{TM} Violet Labeling for Tracking UCB Tregs** 6
Figure S5. **CD3^{+} Tissue Infiltration** 8

Table of Tables

Table S1. **GVHD Score System** 7

Figure S1. ***Phenotypic Characterization of Ex Vivo Expanded UCB Tregs***

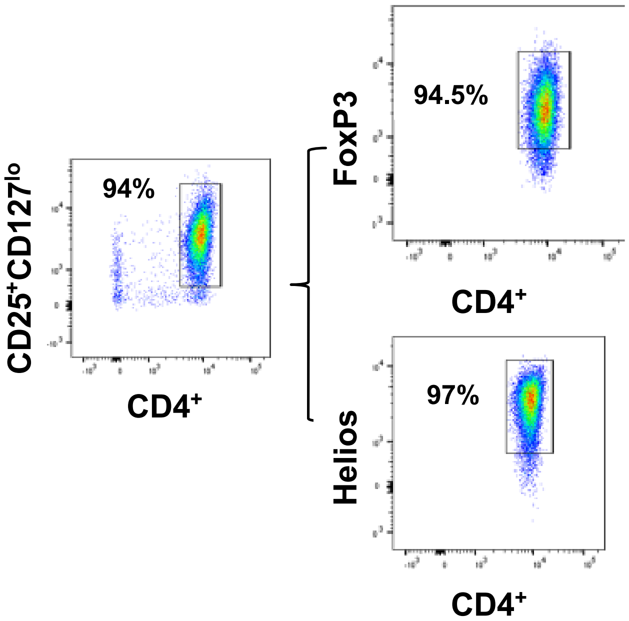

Legend. Phenotypic characterization of UCB Tregs following a 14-day ex vivo expansion. The left panel shows the expression profile of surface markers CD4, CD25, and CD127, identifying a subset of $CD4^{+}CD25^{+}CD127^{lo}$ cells, crucial for Treg identification. The right top and bottom panels display intracellular staining for the transcription factors FOXP3 and Helios, respectively, with high expression levels ($FOXP3^{hi}$ and $Helios^{hi}$), indicative of a regulatory phenotype. These expanded Tregs, characterized by $CD4^{+}CD25^{+}CD127^{lo}FOXP3^{hi}Helios^{hi}$ phenotype, are prepared for subsequent in vitro and in vivo experimental applications.

Figure S2. ***Xenogeneic GVHD model for Ruxolitinib and UCB Tregs***

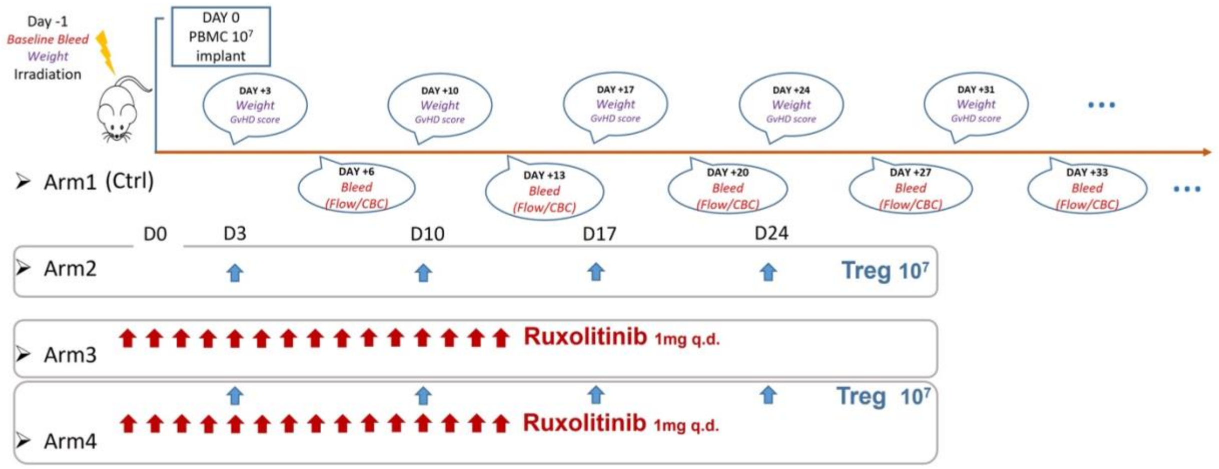

Legend. NSG mice underwent baseline irradiation of 2 Gy one day prior to the experimental interventions. The protocol included administering oral ruxolitinib at 1 mg daily for 14 consecutive days, along with an implantation of 10^7 human PBMCs on the first day. Starting from day 4, mice received weekly intravenous injections of 10^7 UCB Treg cells for four weeks. The experimental design featured several arms: a PBMC arm, a ruxolitinib-only arm, a Treg-only arm, and a combined treatment arm receiving both ruxolitinib and UCB Treg cells. Euthanasia was implemented when weight loss exceeded 20%.

Figure S3. ***Impact of UCB Tregs on Xenogeneic GVL***

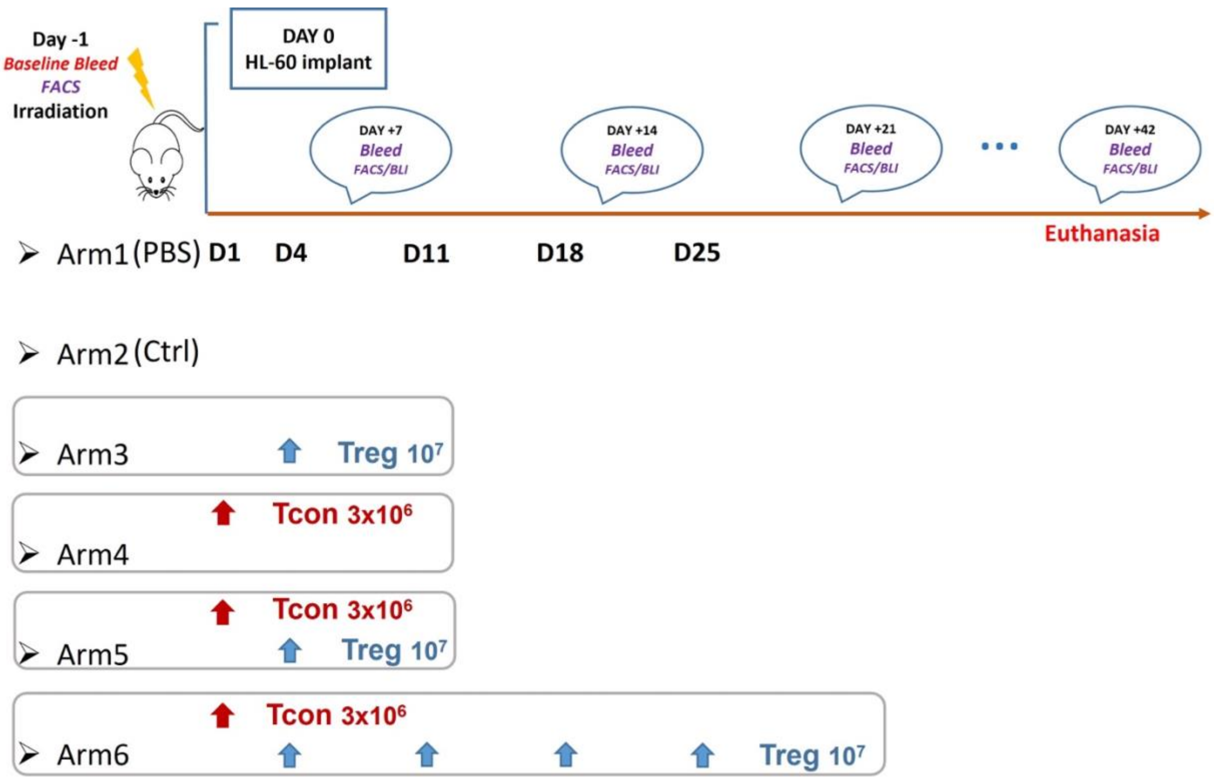

Legend. NSG mice were irradiated with a sublethal dose of 300 cGy one day prior to the study. The experimental setup involved the injection of HL60 GFP-FFluc+ cells, either alone or in combination with 3x10^6 UCB Tregs, CD4+25- Tcon cells, or both. Weekly bleeding and bioluminescent imaging were conducted to monitor tumor progression. The study concluded on day 42 with euthanasia or earlier if a mouse experienced a weight loss exceeding 20%.

Figure S4. ***CellTrace™ Violet Labeling for Tracking UCB Tregs***

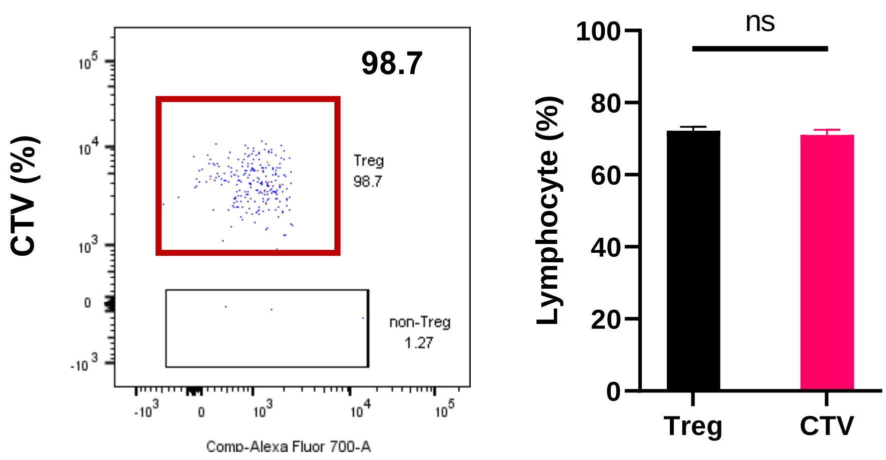

Legend. This figure displays flow cytometric analysis conducted three days after injecting mice with CellTrace™ Violet (CTV)-labeled UCB Tregs. The left panel presents a representative plot of the circulating human cell population (hCD45 stained) in the Treg recipients, demonstrating that nearly all detected cells (98.7%) were CTV-labeled Tregs. The right panel shows the proportion of lymphocytes that are positive for CD4 and CD25 staining compared to those that are CTV-labeled, with no significant difference observed (p = n.s., student t-test). This analysis confirms the utility of CTV for effectively labeling Tregs in vivo.

Table S1. ***GVHD Score System***

Grade	***Weight Loss***	***Posture***	***Activity***	***Fur Texture***	***Skin Integrity***
0	<5%	Normal	Normal	Normal	Normal
1	>5% to <15%	Hunching at rest	Mild to moderate decrease	Mild to moderate rufflings	Scaling of paws and tails
2	>15%	Markedly hunching	Considerable decrease	Marked rufflings	Clearly denuded

GVHD Score based on phenotype characteristics[1].

Figure S5. ***CD3⁺ Tissue Infiltration***

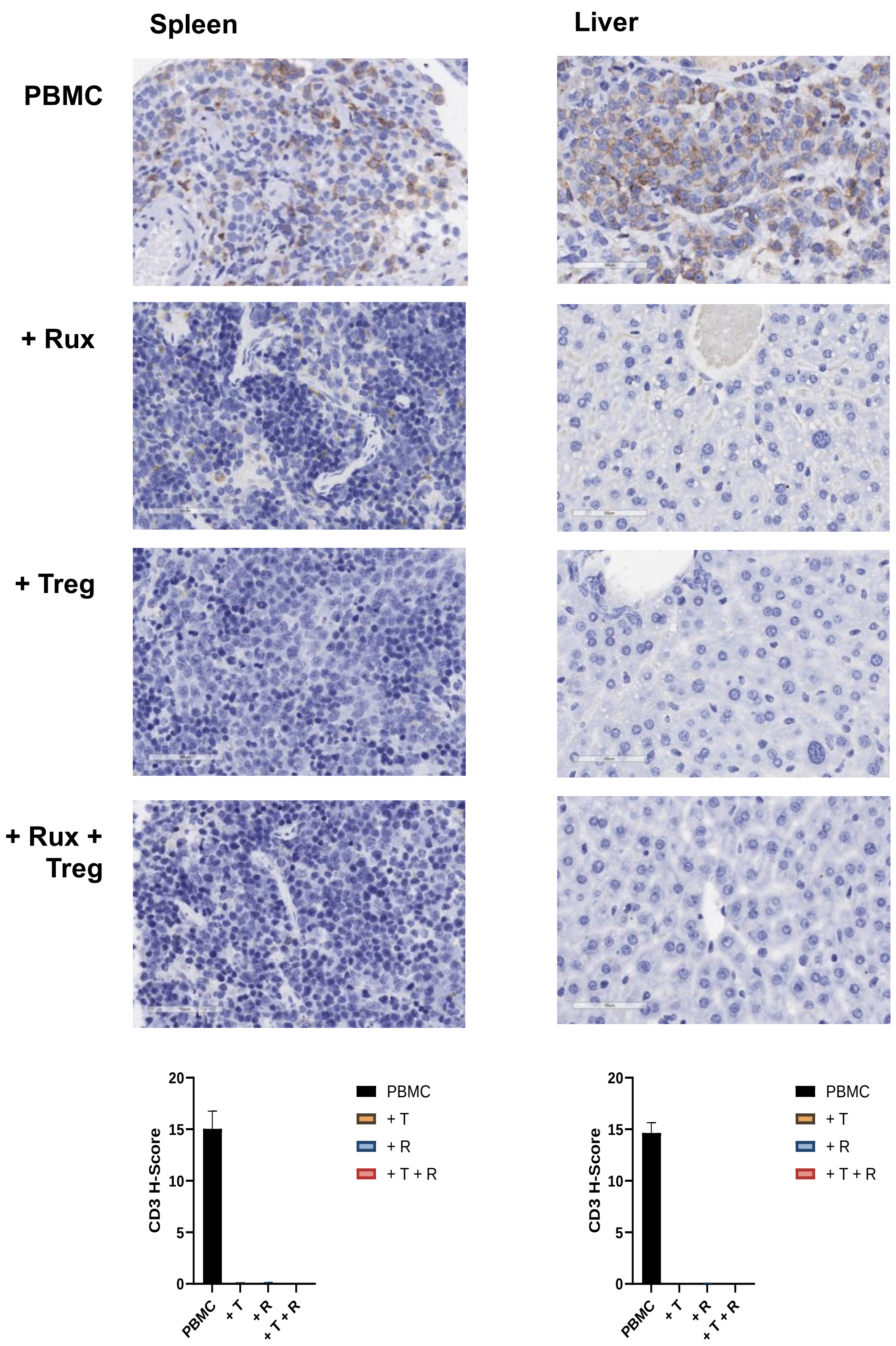

Legend. Histopathologic examinations of spleen and liver at 40x magnification, shows tissue destruction and high $CD3^+$ staining in the control PBMC arm. Tissue architecture is somewhat preserved in PBMC+ruxolitinib arm, with high concentration of $CD3^+$ staining in the alveolar lining as well as in the parenchyma. Complete resolution of $CD3^+$ infiltrate as well as tissue architecture preservation is seen in UCB Treg recipients with or without ruxolitinib. Quantification analysis of the H-score for human CD3 positivity (right panel). The H-score was defined by the percentage of strongly positive stain × 3 + moderately positive stain × 2 + weakly positive stain × 1. A final value of 0–300 was also calculated at 40× magnification using the software HALO (v3.5-3577.140). A $p < 0.05$ was considered statistically significant. * $p < 0.05$, ** $p < 0.01$, *** $p < 0.001$. T= Treg; R=ruxolitinib.

References

1. Reddy, V., et al., *G-CSF modulates cytokine profile of dendritic cells and decreases acute graft-versus-host disease through effects on the donor rather than the recipient.* Transplantation, 2000. **69**(4): p. 691-3.
